# Reduced Lead Exposure Following a Sensitization Program in Rural Family Homes Producing Traditional Mexican Ceramics

**DOI:** 10.29024/aogh.916

**Published:** 2018-07-27

**Authors:** Marcela Tamayo-Ortiz, Jaime Navia-Antezana

**Affiliations:** 1 Consejo Nacional de Ciencia y Tecnología. Avenida Insurgentes Sur 1582, Benito Juárez, Crédito Constructor, 03940 Ciudad de México, D.F., MX; 2 Instituto Nacional de Salud Pública. Universidad No. 655 Colonia Santa María. Ahuacatitlán, Cerrada Los Pinos y Caminera C.P. 62100, Cuernavaca, Morelos, MX; 3 Grupo Interdisciplinario de Tecnologia Rural Apropriada, Mexico Carretera Pátzcuaro a Erongaricuaro No. 28, Tzentzenguaro, C.P. 61613, Pátzcuaro, Michoacán, MX

## Abstract

**Background:**

Traditional ceramics are a cultural heritage in Mexico, used by the general population in everyday life. These ceramics are glazed with lead oxide and are usually produced in households that share living and working spaces. Glazing is usually performed by women, and children are not restrained from the work space and frequently help, resulting in high levels of lead exposure for all. Interventions that promote a change in technology (such as lead-free glazes or efficient kilns) are often unrealistic for potters with fewer economic resources who depend on their production as their main income. Interventions focusing on exposure prevention (rather than a technology change) at the household level are scarce.

**Methods:**

Working hand-in-hand with a group of nine women, lay community workers, *promotoras*, from Santa Fe de Laguna, Michoacán, we developed a program focusing on the self-recognition of health risks. The program was composed of health education (including a lead in blood and bone measurement for women), health/work risk recognition and communication to the community, and work/living area reorganization and remediation in three stages: work with 1) *promotoras*, 2) their extended families, and 3) their community, including talks in elementary schools.

**Results:**

The *promotoras* developed and distributed risk communication graphic materials and delivered a lead-awareness talk in the Purhepecha language, in the local primary health-care clinic and three elementary schools. Lead in bone levels had a mean ± SD (min, max) of rotula: 84.8 µg/g ± 68.9 (23.89, 214.2), tibia 93.2 µg/g 81.2 (14.23, 261.21). We implemented safer and cleaner ceramic production in the *promotoras* workshops.

**Public Health Relevance:**

Environmental and occupational exposures can be reduced through programs that are tailored by and for a specific community. When there is no evident alternative technology for safer production, such programs can empower groups and lead to reduced exposure for their children, family and community.

## Introduction

Traditional earthenware dinnerware is a cultural heritage in Mexico, commonly used by the general population to cook, store and serve food. The production and sale of these consumer products is the main source of livelihood for many artisan families. However, traditional earthenware is frequently coated with lead-based glaze and represents one of the most important sources of lead exposure in Mexico [1,2,3,4]. Traditional artisan workshops often share space with the living space in the family house. This is a result of the work organization, since women are usually in charge of glazing the ceramics as well as cooking and looking after the children. Therefore, it is common to find the kiln, the cooking stove and glazing area all in the same living space, resulting in high levels of lead in the air, soil and house walls [5]. Pregnant women and children are most susceptible to lead, and even low doses of exposure can have neurotoxic effects [6,7,8].

The use of lead-based glazes (lead monoxide also known as *greta* or *litargirio*) is necessary since artisan kilns use wood as fuel and temperatures reached are less than 1000°C. Lead-based glazes have a low melting point and give earthenware a perfect shiny, waterproof finish; however, when in contact with food (particularly acidic food), it will leach lead, even after constant use [9]. A recent animal study showed that lemonade stored in such ceramics had on average 200 µg/L lead, and drinking it resulted in 2.5 µg/dL of lead in the pregnant rats’ blood [10]. For decades, there have been multiple initiatives to solve this problem, which range from normativity and legislation on the lead levels permitted in ceramics, to programs promoting the use of lead-free glazes in artisan workshops.

Despite this, there are many reasons for this pervasiveness, including that artisans with the lowest socio-economic status are those who depend the most on their ceramic production for a living. They are unable to change their kilns (e.g. a kiln using gas or electricity can be unaffordable for an artisan household) or risk using a lead-free glaze that will result in their production not selling. Many artisans have implemented parallel production (lead-free and lead-glazed) often resulting in cross-contamination; others have opted for a different finish such as burnishing, and for some families a change of handcraft has been the solution (like palm weaving). On a population level, risk communication has been limited – one reason for holding back information is the cultural heritage these ceramics represent. Mexican food is often presented and served in these ceramics, even in high-end restaurants. Importantly, health effects of lead are silent and mostly unrecognizable/unidentifiable at an individual level [11].

This article reports the work done in Santa Fe de la Laguna, a rural community of the state of Michoacán, Mexico, where traditional pottery is one of the main economic activities. The purpose of the project was to develop and promote a sensitization and risk assessment program based on local production methods, in order to reduce exposure to lead at a household level. We propose that the implementation of such a program could help empower families producing ceramics by bringing knowledge on the health risks that exposure to lead poses.

## Methods

Between the years 2000 and 2004, the Interdisciplinary Group for Appropriate Rural Technology (GIRA, by its initials in Spanish), a non-government, non-profit organization based in Pátzcuaro, Michoacán, implemented a comprehensive sensitization and risk assessment program for potters. The program integrated exposure assessment, risk management and communication strategies aimed to reduce the occupational exposure to lead in local ceramic workshops. We worked with a group of eight female lay community educators, *promotoras*. This has proven to be a good model for health promotion projects and strategies [12]. Also, in this particular community, women are in charge of the glazing process while men mine clay, collect wood for fuel and load the kiln. All *promotoras* were potters and belonged to the Purhépecha women group UARHI from Santa Fe de la Laguna, Michoacán. The program consisted of 3 stages: work with 1) *promotoras*, 2) their extended families and 3) their community.

*The first stage of the program.* Activities of this stage included work only with the *promotoras* and had 2 main objectives: 1) to understand the health effects of lead and 2) to describe step by step and in detail the ceramic production process and identify occupational risks. We held meetings with potters and their families twice a week, where reading material was shared and discussed with the *promotoras*. The material focused mainly on the health effects of lead and topics on children and reproductive health. The meetings were held in Spanish often with simultaneous translation by one of the *promotoras* to Purhepecha (the native language). We developed a work questionnaire in which each step of the ceramic production was identified and evaluated by the *promotoras*. We included a question in which each *promotora* had to imagine what the best solution for the particular problem in her workshop could be.

*The second stage of the program.* This stage included the *promotoras’* closest family and the main objective was to design and develop effective risk communication strategies in Purhepecha. A list of questions was developed by *promotoras* that they asked their closest families. We then analyzed what activities were common to all the community and what would be the most effective communication media. We developed a calendar that included selected drawings made by the *promotoras* identifying different exposure prevention actions in the workshop and distributed it to their families and closest relatives.

*The third stage of the program.* This stage reached a community level. We designed and developed a talk in Purhepecha that was provided by the *promotoras* in the primary care clinic to women from the community. The talk included children’s and reproductive health topics related to the practice of pottery and simple exposure to lead-prevention strategies. We repeated these four times throughout a month. We then visited four local primary and secondary schools where the *promotoras* gave the talk to children from each school grade. The objective of the talk was that children would be able to identify risks derived from helping their parents in pottery production and how to prevent lead exposure.

### Lead in venous blood and bone measurements

In May 2002 lead concentrations in blood and bone were measured in the *promotoras* and their main organizer in the community. This was a collaboration with the National Institute of Public Health at the American British Cowdray Hospital’s (ABC Hospital) environmental health research center in Mexico City. Lead in bone has a half-life of up to 30 years (indicating chronic exposure), whereas in blood, it is about 25 days (showing recent/acute exposure) [13]. Lead in bone was measured in the cancellous (patella) and cortical (tibia) bone using a K-shell X-ray fluorescence instrument (KXRF). Each leg was measured for 30 minutes, and the results from both legs were averaged, weighted by the inverse of the measurement variance. Lead in blood was drawn into royal blue trace metal vacutainer (Becton-Dickinson and Company, Franklin Lakes, New Jersey) tubes containing EDTA. Lead concentrations were measured using a dynamic reaction cell inductively coupled plasma mass spectrometer (Elan 6100; PerkinElmer, Norwalk, CT) at the laboratory of the ABC Hospital.

Participation in this program was voluntary and the *promotoras* were awarded a small monthly monetary compensation. The program was funded by the North America Fund for Environmental Cooperation (Fondo de América del Norte para la Cooperación Ambiental, FANCA,) project number 2001125, and the Mexican Fund for Nature Conservation (Fondo Mexicano para la Conservación de la Naturaleza, FMCN), project number G1-01/017.

## Results

*The first stage of the program.* Knowledge of the negative health effects of lead as well as basic concepts of reproductive processes were limited in our working group. A series of health hazards related to the use of lead glazes and risks associated with work and living spaces were identified by the *promotoras* from the moment the glaze is purchased throughout the production process.

*Glaze purchase. Greta* is a bright red or yellow powder and can be purchased by the pound in local convenience stores, directly from the sack which can be next to fresh produce. It is handled manually by the shop attendant, weighed in the same balance as fruits or vegetables, and packed in plastic bags that people (usually women) bring home together with other food items purchased.

*Glaze preparation and use. Greta* is mixed with water in plastic tubs without any gloves since this would affect the sensibility of the hand to the correct thickness of the glaze. The ceramic vessel is dipped in the mix to cover it and placed on the side. This procedure is usually carried out on the floor (often dirt), sometimes sitting on a woven palm rug, and results in glaze spilling on the surrounding area (accumulating over time). Cooking areas are shared and in many of the workshops there is a small wood-fire stove close by, such that the woman can be both glazing and cooking at the same time. While glazing, a woman usually wipes her hands on her skirt to attend other activities, including carrying or breastfeeding her child.

*Cleaning of leftover glaze and work utensils*. Once the glazing is done, the same plastic tubs where the glaze was mixed are used to wash cooking and eating utensils. Often there is only one water tap available per household and the lead-contaminated water will be disposed of in the same drainage.

*Health Promotion Strategies*. With the results obtained in the first stage of the program we implemented strategies that could reduce lead exposure at the household level for the *promotoras* and their families.

*For the promotoras and their families*. We designed and made aprons that were designed specifically long enough to cover ankle-long skirts and easy to put on and remove. The *promotoras* and their families planned a new work/living space layout. We changed the dirt floor for a concrete one in their houses and organized the work space such that it was as separate as possible from the living areas. Where possible, we installed shelves and provided new plastic tubs, exclusive for glazing. In each of the *promotoras’* houses/workshops, a water system with three taps was installed, such that water used in the pottery production did not interfere with the cooking or cleaning. A separate drainage was also installed.

**Figure 1:**
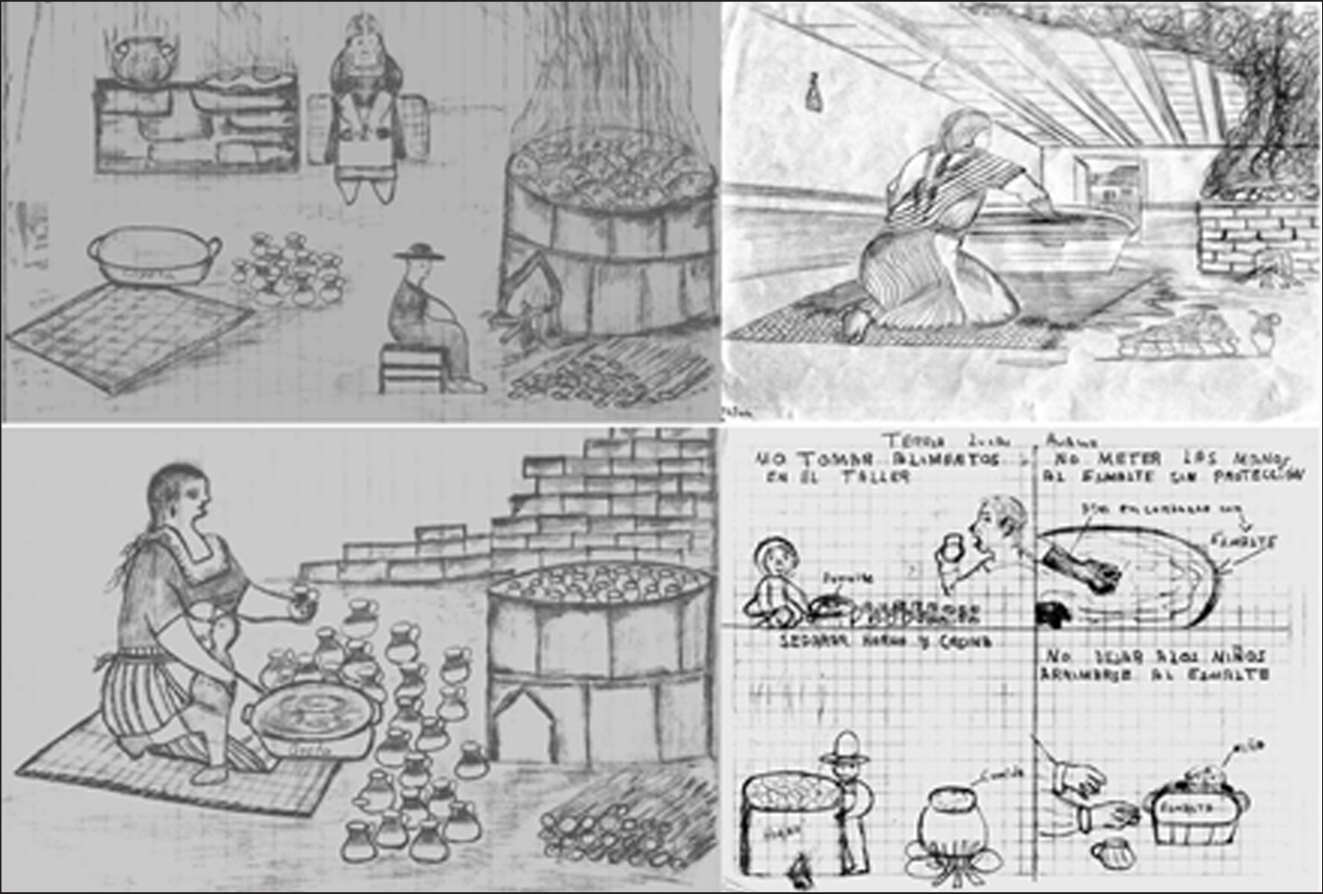
Drawings created by the *promotoras* depicting their living/work areas.

**Figure 2:**
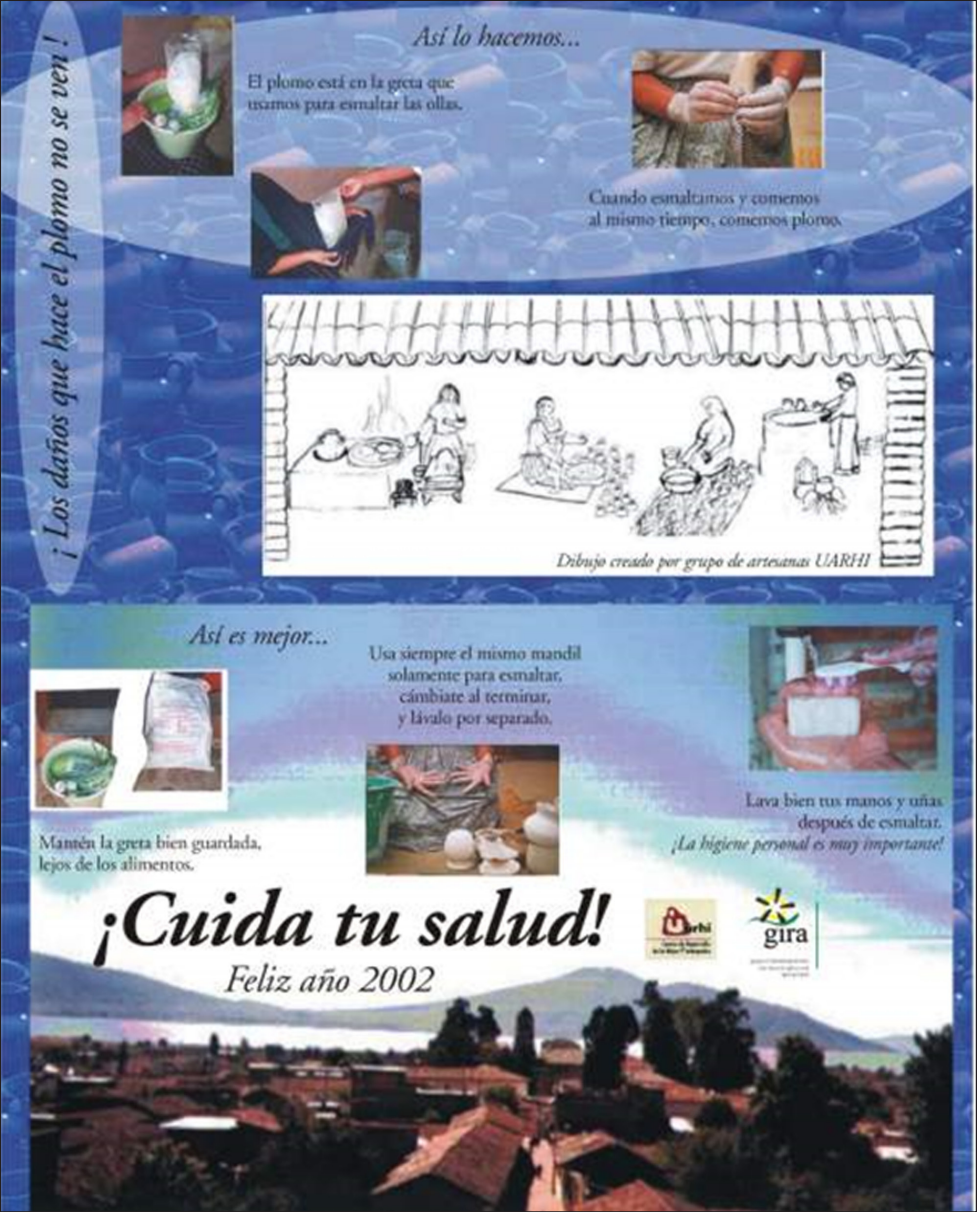
Calendar designed by *promotoras* distributed to their closest family and relatives.

*The second stage of the program (for promotoras’ closest family).* One of the main challenges we faced as a group was to adequately communicate the health risk and exposure prevention messages to the *promotoras’* families and closest relatives. Photographs and drawings (Figure 1) were preferred over text. A calendar was identified as the best printed strategy for the community, since they are very popular. We designed a printed year calendar that was distributed to the *promotoras’* closest extended families (Figure 2). The calendar included images (photographs and drawings) made by the *promotoras* of the main health-protection messages, “This is how we do it” and “This is what we can do”, such as washing hands after using *greta* and before cooking or feeding a child and, when purchasing *greta*, carrying it separately to avoid contamination.

**Figure 3:**
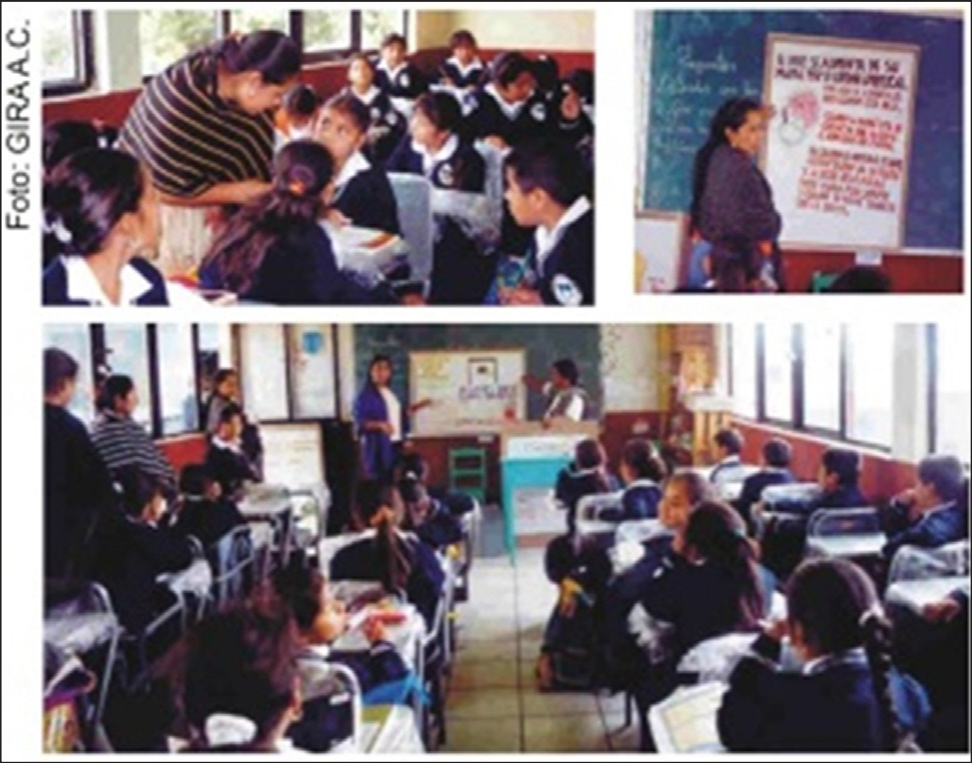
*Promotoras* giving health talks in local schools.

*The third stage of the program (for the community).* The *promotoras* gave a health talk they designed with visual aids, such as flip boards, to approximately 100 women in the primary care clinic and in three primary and middle schools to approximately 500 children (Figure 3). The talk was specifically designed in Purhepecha, and the main messages were specific to health and avoiding exposure to lead. However, much care was taken to avoid a message forbidding the use of lead glazes.

### Results from Lead in Bone and Blood Tests

In May 2002, the mean age of the *promotoras* was 31 ± 8.5 years with the youngest being 15 and the oldest 40. The mean blood lead level was 14.8 ± 8.9 µg/dL, the lowest being 6.7 µg/dL and the highest 29.7 µg/dL. As for lead in bone results, cancellous bone had a mean concentration of 84.8 ± 68.9 µg/g (min 23.89 µg/g, max 241.20 µg/g) and cortical bone a mean concentration of 93.2 ± 81.2 µg/g (min 14.23 µg/g, max 261.21 µg/g) (Table 1).

**Table 1:** Lead concentrations in blood and bone for women in a Mexican rural community of traditional potters.

	Age	Blood µg/dL	Cancellous bone (patella) µg/g	Cortical bone (tibia) µg/g

**Mean ± SD**	30.7 ± 8.5	14.8 ± 8.9	84.8 ± 68.9	93.2 ± 81.2
	15	11.6	24.26	14.23
	21	7.6	75.06	76.11
	28	19.3	83.99	101.03
	28	13.1	64.98	82.19
	33	12	23.89	33.06
	35	27.9	214.20	261.21
	37	29.7	160.95	150.17
	39	5.8		
	40	6.7	31.30	27.49

**Figure 4:**
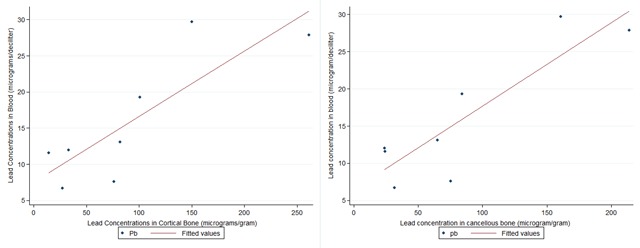
Association between lead concentrations in blood and cancellous bone (left) and cortical bone (right).

Lead concentrations in blood and bone were strongly correlated: between lead in blood and cancellous bone of r = 0.88, lead in blood and cortical bone of r = 0.84 and between cancellous and cortical bone of r = 0.98. Figure 4 shows these associations, all of which were statistically significant (p value < 0.05).

## Discussion

An important characteristic of this sensitization program was that our main objective was not the substitution of *greta* (lead-based glaze); it was rather identifying the different occupational risks derived from the production of traditional ceramics. This was a factor that was taken care of carefully throughout our work with the *promotoras* and later with their families and community, since our program was not the first that had been implemented in the community. The Mexican government agency Fondo Nacional para el Fomento de las Artesanías, FONART (Mexican Fund for Handcrafts) has implemented programs throughout Mexico to remove lead-based glazes since the early 1990s [14]. However, the change to lead-free glazes does not only involve their use, but other factors that include: a better kiln that can reach uniform temperatures, remediation of the workshops and a market for lead-free ceramics. Also, at the time our program was implemented, lead-free glazes were not as developed as in more recent years [15]. Artisans that had used lead-free glazes often reported that their products had a milky finish to them, making them unsellable. For a traditional workshop, where every person in the family is involved in the process, producing a full kiln of ceramics can take many weeks. In many cases the glazing with *greta* will be last in a long chain of steps that start in a clay mine, then grinding, mixing and preparing the clay, molding and shaping the clay pieces (when the clay is wet), letting the piece dry, a first fire in the kiln, decorating, and finally the last coat of glaze (*greta*) before a second firing in the kiln for the end product. Risking the entire production is not a possibility, especially for the most marginalized families. For example, this particular community has a high rate of migration of men to the US. It is not uncommon to find households where a woman and her children are entirely dedicated to the production of ceramics. Working with women was a second important characteristic of our program, and all of them were potters. Therefore, during the first stage of the program, it was crucial that the *promotoras* understood the health hazards that they and their families were exposed to through the use of lead-based glazes.

Unlike other programs that have been implemented, we worked closely and frequently with the group of women from the community. During the bi-weekly meetings, health themes that would be reviewed were as detailed as the group would inquire. This allowed us to answer many questions on reproductive health, pregnancy and child development that enabled the understanding of the toxicity of lead. Although all the *promotoras* speak Spanish, the local language is Purepecha. Since the group was composed of only women (including the staff member from GIRA) and we were able to include a translator, this helped the working dynamic and generated an environment of trust between women. This empowered the *promotoras* and enabled the talks in the community health center and the schools.

The results from the lead concentrations in blood and bone were striking for the *promotoras*; those who had higher lead concentrations were also those that produced more ceramics. Although the results are from a small group of women, the concentrations found in the blood are higher than the recommended level of 10 µg/dL for women occupationally exposed, according to Mexican normativity [16]. Regarding lead in bone, the results are also higher that those found in a group of 41 women in Boston, who had a mean concentration of lead in cancellous bone of 5.8 ± 4.5 μg/g and of 4.5 ± 4.0 μg/g in cortical bone [17]. In a Mexican cohort of women, mean lead concentrations one month after birth were 14.24 ± 14.19 μg/g for cancellous bone and of 9.67 ± 9.21 μg/g for cortical bone [18]. Some of the results were above 100 μg/g, which points at a chronic elevated exposure. In a study done in the same community, Hibbert et al. [5] measured lead in personal air and found concentrations were up to 454 μg/m^3^ for workers performing pottery firing and glazing (The NIOSH recommended exposure limit is a time weighted average of 50 μg/m^3^ over 8 hours). Concentrations measured in soil lead of 17 homes ranged from 0.39 to 19.8 μg/m, and dust lead on surfaces of household items, hands and clothes of an artisan ranged from 172 to 33,060 μg/f^2^ [5]. All of the women were in their reproductive stage, and had young children living at home. A pooled analysis by Lanphear at al. [19] demonstrated that lead in dust was the major source of lead exposure for children. However, in their same study, Hibbert et al. [5] found that the main source of exposure for children living in Santa Fe de la Laguna was food cooked in earthenware glazed with *greta* (a content of 2.4 ppm of lead was found in food cooked in such a pot; as a reference, the FDA has established a limit of 0.1 ppm of lead for food to be eaten by children) [20].

We are aware that considerable time has elapsed since the implementation of our program and this report; however, lead-glazed ceramics are still being produced, sold and used in Mexico. They are the main source of exposure to lead for the general population [4]. Today, one of the main challenges Mexico still faces is completely phasing out the use of leaded glazes while preserving the cultural inheritance of traditional ceramics. Awareness to consumers is not an option, unless there is a clear method to distinguish lead-free earthenware. In fact, a difficult task in raising awareness of lead’s toxicity is that its effects most likely cannot be noticed by eating food in lead-glazed earthenware. A more efficient method to protect the population would be to ensure that all traditional earthenware produced is lead-free, a silent change, without affecting the artisans’ sales.

## Conclusions

The sensitization program presented here has a broader spectrum than lead exposure. Artisans who have a better understanding of the health hazards that their occupation implies are more likely to 1) avoid occupational exposure and 2) be more open to new and alternative technologies (such as lead-free glazes or alternative energy kilns). Our program was not designed as an epidemiologic study, nor was there a formal evaluation of its possible impact; however, at least two of the *promotoras* that participated in the program have switched permanently to a lead-free production. The use of lead-free glazes goes beyond health or technology, it implies having the support of the potters’ sector working together with all actors involved in the change.
